# A Needle in the Fetal Brain: The Rare Role of Transabdominal Cephalocentesis in Fetal Hydrocephalus

**DOI:** 10.7759/cureus.14337

**Published:** 2021-04-07

**Authors:** P Swetha, Shobha Dhananjaya, Amogh Ananda Rao, Ashutosh Suresh, Chiranth Nadig

**Affiliations:** 1 Obstetrics and Gynaecology, Jagadguru Jayadeva Murugarajendra Medical College, Davangere, IND; 2 Internal Medicine, Jagadguru Jayadeva Murugarajendra Medical College, Davangere, IND

**Keywords:** fetal hydrocephalus, transabdominal cephalocentesis

## Abstract

Fetal hydrocephalus is a fairly common occurrence in pregnant women, surfacing early or late in the pregnancy. The perinatal and pediatric outcomes are largely determined by the cause of hydrocephalus and the extent of the irreversible destruction of the brain tissue. In pregnancies where the fetal prognosis is unfavorable, aspirating the cerebrospinal fluid (CSF) to facilitate vaginal delivery is an option.

In this report, we present the case of a primigravida with term fetal hydrocephalus who underwent ultrasound-guided transabdominal cephalocentesis and subsequently delivered vaginally without any adverse perinatal outcomes.

## Introduction

Fetal hydrocephalus is not uncommon in the practice of obstetrics, with an incidence of around 0.2-1 per 1,000 live births [1]. Hydrocephalus can be broadly classified into two types based on its etiology: congenital and acquired. Generally, developmental malformations like the Arnold-Chiari and Dandy-Walker malformations, megalencephaly syndromes, and the VACTERL-H spectrum are associated with congenital hydrocephalus. Infections and hemorrhage represent the other etiological factors causing hydrocephalus [2]. Its pathobiology usually involves the obstruction of the ventricular system, most frequently occurring at the aqueduct of Sylvius. The perinatal and pediatric outcomes are largely determined by the cause of hydrocephalus and the extent of the irreversible destruction of the brain tissue. In pregnancies where the fetal prognosis is unfavorable, aspirating the cerebrospinal fluid (CSF) to facilitate vaginal delivery is an option. Cephalocentesis is a procedure to drain the excess CSF from the fetus. It is commonly performed vaginally or through the abdomen with ultrasonographical guidance. This method averts the morbidity that ensues obstructed labor, and a cesarean section, especially in primigravida.

We discuss the case of a primigravida with term fetal hydrocephalus who underwent ultrasound-guided transabdominal cephalocentesis and subsequently delivered vaginally without any adverse perinatal outcomes.

## Case presentation

A 19-year-old primigravida with 37 completed weeks of gestation was referred to our center in September 2020 due to fetal hydrocephalus for further management. Her term scan had revealed a single, live, intrauterine pregnancy of around 36 weeks' gestation in the longitudinal lie, with gross hydrocephalus. The placenta was located in the fundal left-lateral region. She had no comorbid conditions during the antenatal period and had followed up regularly with the obstetrician.

**Figure 1:**
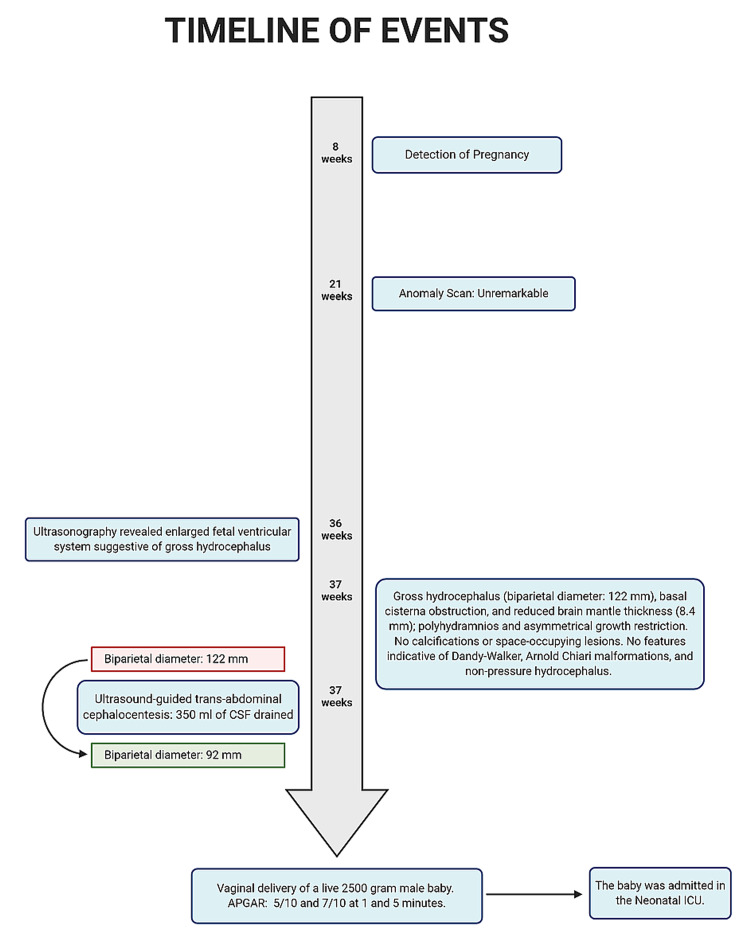
Timeline of events CSF: cerebrospinal fluid; ICU: intensive care unit; APGAR: Appearance, Pulse, Grimace, Activity, and Respiration

Three ultrasound scans were done during the 14th, 17th, and 21st weeks of gestation; all the scans were unremarkable (Figure 1). On detailed ultrasonographic examination at 37 weeks of gestation, we found an enlarged fetal cerebral ventricular system suggestive of hydrocephalus (biparietal diameter: 122 mm), basal cisterna obstruction, and reduced brain mantle thickness (8.4 mm). The estimated fetal weight was 2,500 grams. No calcifications or space-occupying lesions were noticed. No features indicative of Dandy-Walker and Arnold Chiari malformations, or non-pressure hydrocephalus were noticed. However, we observed polyhydramnios and asymmetrical growth restriction.

Infectious etiology of hydrocephalus was ruled out on obtaining negative results for TORCH panel, HIV, hepatitis B, tuberculosis, and also coronavirus. The neonatologist opined that the prognosis of the baby is guarded due to cerebral atrophy.

After counseling the parents regarding the poor perinatal outcome and the plausible risks of a ventriculoperitoneal shunt procedure following cesarean delivery, the mother opted for a vaginal delivery. Therefore, we planned the vaginal delivery at 37 weeks of gestation to avoid the potential maternal morbidity associated with a primary cesarean section besides affirming the mother’s wish. The patient had neither set into spontaneous labor nor was the fetal head engaged. Consequently, we counseled the patient for ultrasound-guided transabdominal cephalocentesis, and she consented to it despite its invasive nature.

**Figure 2:**
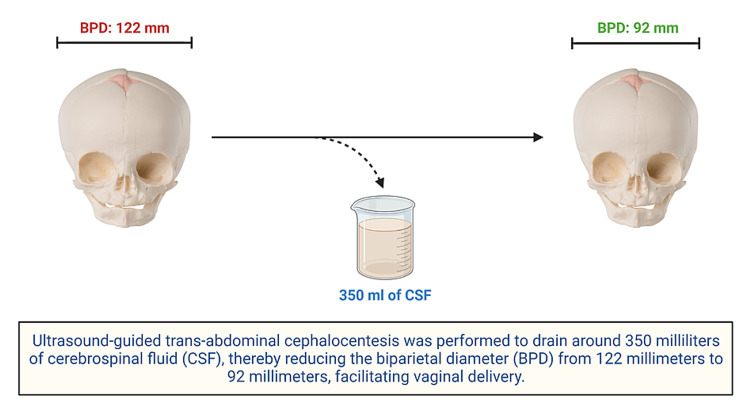
Representative illustration of the reduction in biparietal diameter BPD: biparietal diameter; CSF: cerebrospinal fluid

**Figure 3:**
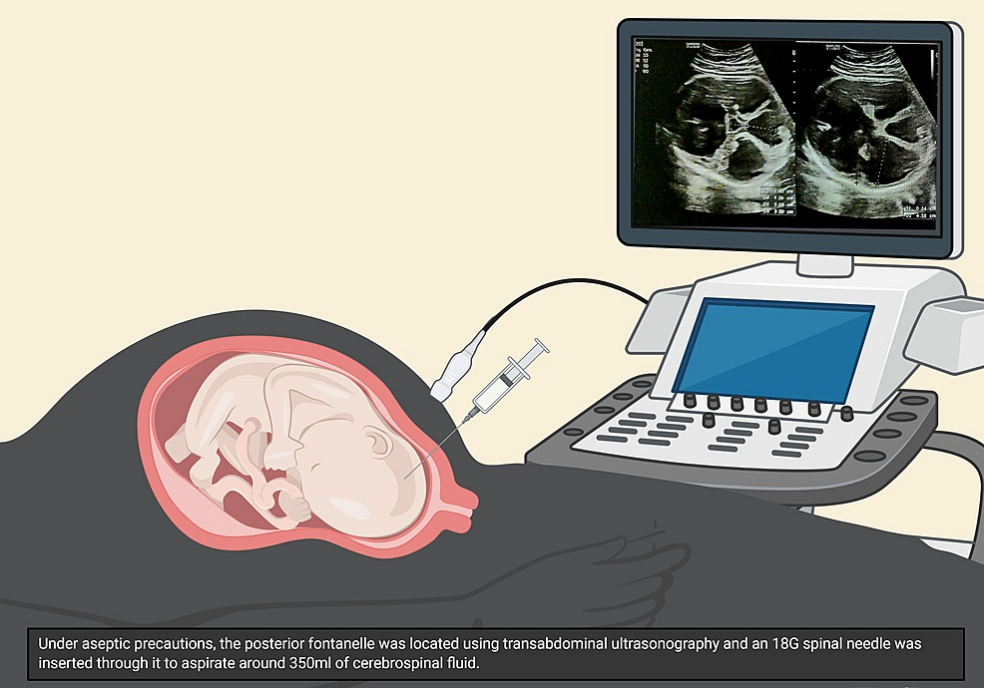
Representative illustration of the procedure

Local anesthesia was administered under aseptic precautions. The posterior fontanelle was located using transabdominal ultrasonography. An 18-gauge spinal needle was inserted through the posterior fontanelle and aspirated to ensure the free flow of clear fluid. Around 350 milliliters of CSF was drained. The biparietal diameter reduced significantly from 122 mm before the procedure to 92 mm after draining the CSF (Figures 2, 3).

The patient was induced with a single dose of 25-mcg oral misoprostol and further augmented with intravenous low-dose oxytocin titration. The first and second stages of labor progressed satisfactorily and she delivered a live 2,500-gram male baby vaginally. The baby cried immediately after birth and had an Appearance, Pulse, Grimace, Activity, and Respiration (APGAR) score of 5/10 and 7/10 at one and five minutes, respectively. The baby was admitted to the neonatal ICU for three days, but the parents were unwilling to have the child undergo further medical intervention, for the prognosis was guarded. The baby expired two months after birth due to intractable seizures. A fetal autopsy could not be performed as the parents did not give consent to it.

## Discussion

Hydrocephalus, the enlargement of the fetal ventricular system, is associated with a repertoire of conditions. Antenatally, on ultrasonography, a separation of more than 33 mm between the ventricular margin and the choroid plexus is considered abnormal; macrocephaly is an occasional finding. As a result of the increased intracranial pressure, there is usually a variable degree of parenchymal thinning [3]. The overall incidence of fetal hydrocephalus is around 0.2-1 per 1,000 live births [1].

Factors such as the gestational age at the time of diagnosis, fetal viability, and the association with other malformations primarily dictate the obstetric management of hydrocephalus. Besides an eventual cesarean section, management options include the placement of a ventricular-amniotic shunt or cephalocentesis [4]. Decompression of the fetal skull can be performed either transabdominally under ultrasound guidance or transvaginally. Since the transabdominal route is more invasive and painful, the latter is preferred due to its psychological advantage [5]. Chasen et al. have reported in their study that perinatal death occurred in 10 of the 11 cases where cephalocentesis was performed [6]. In an analysis of 87 fetal hydrocephalus cases, Garne et al. reported that 53% of the fetuses were still-born or were terminated [7]. However, intractable seizures and global developmental delay with brainstem dysfunction occurred in the majority of survivors [6]. Ventricular-amniotic shunting, when performed in cases of obstructive hydrocephalus, was associated with severe neurological morbidity and procedure-related fetal mortality [8].

Although transabdominal cephalocentesis is a destructive procedure, the physician's beneficence-based obligation to the mother in avoiding a cesarean section must be considered in these cases.

## Conclusions

Even though cephalocentesis is a destructive procedure and not in routine use, it still has a pertinent role in modern obstetrics practice. Cephalocentesis is a worthy alternative to a cesarean section in circumstances where the fetus is nonviable or has a rather dismal prognosis after birth.
